# Doxycycline prevents blood–brain barrier dysfunction and microvascular hyperpermeability after traumatic brain injury

**DOI:** 10.1038/s41598-022-09394-4

**Published:** 2022-03-30

**Authors:** Bobby D. Robinson, Claire L. Isbell, Anu R. Melge, Angela M. Lomas, Chinchusha Anasooya Shaji, C. Gopi Mohan, Jason H. Huang, Binu Tharakan

**Affiliations:** 1 Department of Surgery, Baylor Scott and White Medical Center, Baylor Scott and White Research Institute, Temple, TX USA; 2 Texas A&M University Health Science Center College of Medicine, Temple, TX USA; 3 Amrita Center for Nanosciences and Molecular Medicine, Kochi, Kerala India; 4 Department of Neurosurgery, Texas A&M University Health Science Center College of Medicine, Temple, TX USA; 5 Present Address: Department of Surgery, Morehouse School of Medicine, 720 Westview Dr, Atlanta, GA 30310 USA

**Keywords:** Vascular diseases, Diseases of the nervous system

## Abstract

The main objective of this study was to determine the cellular and molecular effects of doxycycline on the blood–brain barrier (BBB) and protection against secondary injuries following traumatic brain injury (TBI). Microvascular hyperpermeability and cerebral edema resulting from BBB dysfunction after TBI leads to elevation of intracranial pressure, secondary brain ischemia, herniation, and brain death. There are currently no effective therapies to modulate the underlying pathophysiology responsible for TBI-induced BBB dysfunction and hyperpermeability. The loss of BBB integrity by the proteolytic enzyme matrix metalloproteinase-9 (MMP-9) is critical to TBI-induced BBB hyperpermeability, and doxycycline possesses anti-MMP-9 effect. In this study, the effect of doxycycline on BBB hyperpermeability was studied utilizing molecular modeling (using Glide) in silico, cell culture-based models in vitro, and a mouse model of TBI in vivo. Brain microvascular endothelial cell assays of tight junction protein immunofluorescence and barrier permeability were performed. Adult C57BL/6 mice were subjected to sham versus TBI with or without doxycycline treatment and immediate intravital microscopic analysis for evaluating BBB integrity. Postmortem mouse brain tissue was collected to measure MMP-9 enzyme activity. It was found that doxycycline binding to the MMP-9 active sites have binding affinity of −7.07 kcal/mol. Doxycycline treated cell monolayers were protected from microvascular hyperpermeability and retained tight junction integrity (*p* < 0.05). Doxycycline treatment decreased BBB hyperpermeability following TBI in mice by 25% (*p* < 0.05). MMP-9 enzyme activity in brain tissue decreased with doxycycline treatment following TBI (*p* < 0.05). Doxycycline preserves BBB tight junction integrity following TBI via inhibiting MMP-9 activity. When established in human subjects, doxycycline, may provide readily accessible medical treatment after TBI to attenuate secondary injury.

## Introduction

Traumatic brain injury (TBI) is a significant and global public health concern affecting more than 57 million annually^1^. Studies show that in the United States, more than5 million people live with a TBI-related disability^2^. TBI patients with severe neurological dysfunction typically have a poor quality of life and considerable financial problems^3^. The exact expenses associated with TBI are mostly undercalculated, and do not account for the years of lost income and ailment^4^. TBI can lead to devastating neurological effects and will primarily involve in the major impairment or death of a person. The near future^1^. The TBI patient care is limited, with one of the main difficulties being to effectively address the adverse consequences of cerebral edema that leads to an elevation of intracranial pressure (ICP). There are no effective therapies or approaches to alleviate the symptoms of TBI by addressing the underlying pathobiology.

At a cellular and molecular level, TBI causes major and minor injuries. This in turn leads to the formation of reactive oxygen species along with the activation of different proinflammatory cytokines and several proteolytic enzymes.

In TBI, depending on the severity of the impact, primary injury may occur as direct tissue damage from the impact, resulting in irreversible tissue/cellular and damage to the blood vessel at various levels. This leads to a series of biological events leading to secondary injuries. The damage caused due to this phenomenon might be through host cellular response or changes occurring by corresponding physiological effects. During the phase of the secondary injury, abnormalities in the blood–brain barrier (BBB) can result in various derangements that eventually leads to a patient’s adverse clinical outcomes. These mechanisms cause the loss of BBB integrity and normal microvascular permeability of the barrier^5–7^. The BBB is a semipermeable membrane with a highly complex structure, consisting of inter-endothelial tight junctions and adherens junctions, and other cellular and matrix components of the neurovascular unit, for the preservation of the brain parenchyma and cerebrospinal fluid^7–10^. The deleterious cellular cascade of events in TBI results in microvascular hyperpermeability of the BBB^5–7^. This hyperpermeability leads to cerebral edema and injury and death of neurons and other cell types^11,12^. Identification of novel therapeutic targets and development of drugs, against secondary injury by preserving the BBB integrity could be revolutionary in the care of head trauma patients.

Matrix metalloproteinases (MMPs) belongs to a family of endopeptidases that collectively cleave the constituents of the extracellular matrix. MMP-9 is a ubiquitous proteolytic enzyme that play a significant role in new blood vessels formation (angiogenesis), wound healing, and embryonic development, along with a litany of other cellular and physiological processes. In the brain, MMP-9 is present at relatively low levels in normal conditions but significantly increases its expression and activity acutely following TBI^13,14^. Enhanced MMP-9 enzymatic activity leads to damage of the BBB tight junctions by targeting the tight junction proteins resulting in barrier dysfunction and further neuroinflammation^10,15^. Doxycycline, a tetracycline antibiotic and known for its broad-spectrum coverage, has been shown to inhibit MMP-9 activity in traumatic processes such as in thermal injury^16–18^ but its potential effect on BBB breakdown and brain edema is not known^19^. Doxycycline is lipophilic, making it readily absorbed after oral ingestion, has rapid accessibility through intravenous injection, and possesses BBB crossing ability^20^. The main objective of the present study was to understand the therapeutic potential of doxycycline against TBI-induced BBB breakdown and microvascular hyperpermeability. We tested the hypothesis that doxycycline possesses anti-MMP-9 activity and will attenuate its activity in the brain and protect the BBB against the MMP-9-mediated tight junction breakdown, following TBI.

## Results

### In silico MMP-9 and doxycycline binding analysis

**Figure 1:**
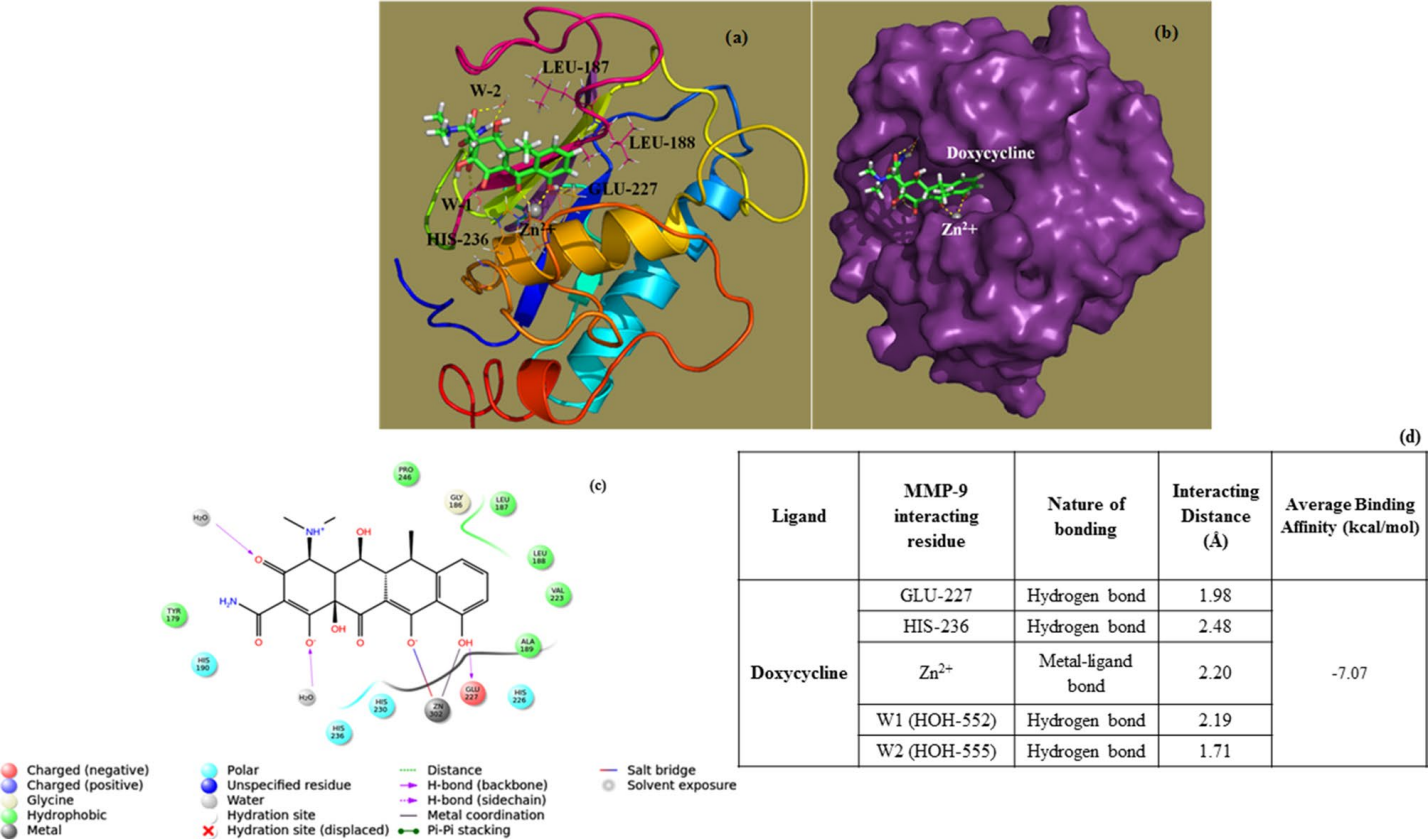
Molecular docking studies demonstrating doxycycline interaction with MMP-9 protein (PDB ID: 4XCT). Doxycycline interacting with active site residues of MMP-9 protein shown in sticks representation (**a**). MMP-9 surface map and the binding of Doxycycline in its active site cleft (**b**). 2D-interaction diagram of Doxycycline with MMP-9 protein showing hydrogen bonding, metal–ligand and hydrophobic interactions (**c**). Table quantifying and describing molecular docking based atomic level interactions between MMP-9 active site residues and doxycycline (**d**).

Glide docking showed favorable interactions between MMP-9 enzyme active site residues and doxycycline. These chelating groups bind to Zn^2+^ ion thus preventing its contribution for enzyme activity. Similarly, present docking results support the experimental evidence of MMP-9 inhibition by Zn^2+^ ion chelation. The glide score which reveals the binding affinity of MMP-9 with doxycycline was −7.097 kcal/mol. A binding affinity less than 0 kcal/mol denotes a thermodynamically favorable reaction. Here the carboxyl and hydroxyl groups of doxycycline chelate catalytic Zn^2+^ ion and thereby inhibit the MMP-9 enzyme's activity (Fig. 1a, b). The two-dimensional (2D) interaction diagram shows different types of interactions which include hydrogen bonding, hydrophobic, and metal ion coordination between MMP-9-drug complexes. GLU-227 and HIS-236 make key hydrogen bonding interactions with doxycycline along with the Zn^2+^ ion binding. Further, TYR-179, LEU-187, LEU-188, ALA-189 and PRO-246 of MMP-9 make hydrophobic interactions with doxycycline (Fig. 1c). The hydrogen bonding interacting residues and its distances were calculated (Fig. 1d).

### Doxycycline protects tight junction integrity in vitro

**Figure 2:**
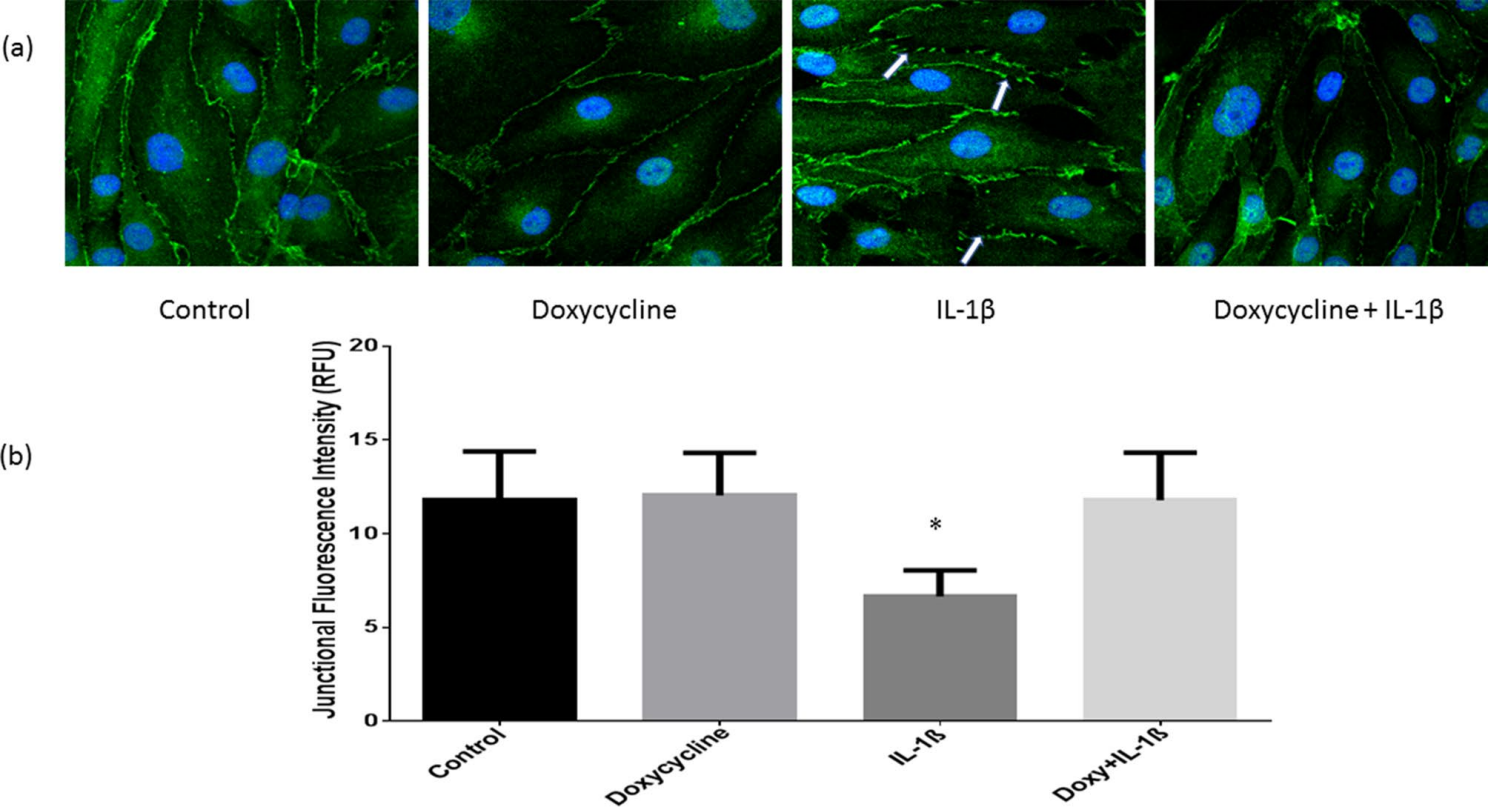
Evaluation of tight junction integrity by immunofluorescence of ZO-1 in the tight junctions. Rat brain microvascular endothelial cells were exposed to IL-1β and/or pre-treated with doxycycline. (**a**) Localization of ZO-1 at areas of cell-cell contacts/tight junctions. Arrows indicate areas of tight junction disruption. (**b**) Quantification of ZO-1 immunofluorescence. There was significant decrease in the ZO-1 localization in the cells treated with IL-1β (n = 4). This was prevented with treatment of doxycycline (n = 4). The analysis was performed with ImageJ. Compared to control *p < 0.05. Compared to IL-1β **p < 0.05.

In endothelial cells, the inter-endothelial tight junctions and the tight junction associated proteins play a major role in the tight junction integrity maintenance in the areas of cell–cell contacts. The immunofluorescence localization of tight junction-associated proteins is helpful for determining barrier integrity and tight junction organization. The imaging data obtained from the immunofluorescence of ZO-1 in rat brain microvascular endothelial cells is shown (Fig. 2a). The result shows that in untreated control cells, ZO-1 is localized continuously at cell–cell junctions whereas IL-1β (10 ng/mL: 2 h) treatment led to decreased signal/localization at the tight junctions. Doxycycline treatment (20 µM; 1 h) prior to IL-1β showed retention of ZO-1 at the inter-endothelial tight junctions (Fig. 2a). Figure 2b is the quantification of the fluorescence signal representing ZO-1. There was a significant decrease of immunofluorescence signal of the cells treated with IL-1β and doxycycline treatment attenuated this effect significantly (*p* < 0.05; n = 4).

### Monolayer hyperpermeability is decreased with doxycycline in vitro

**Figure 3:**
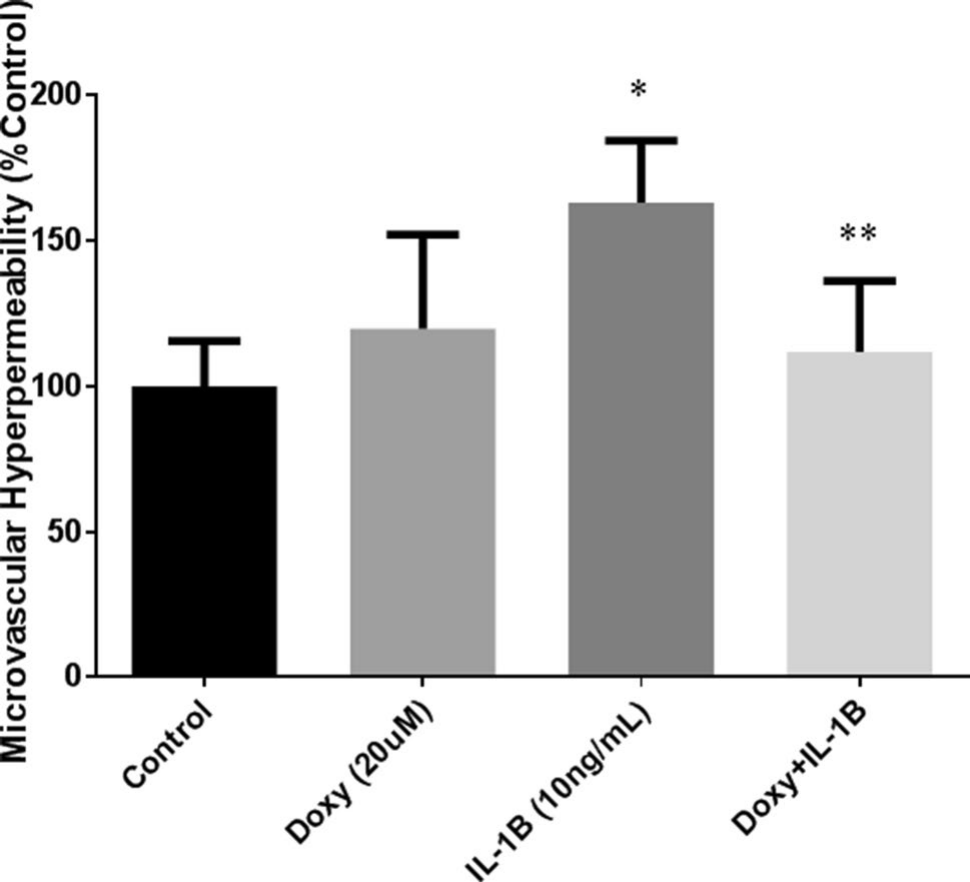
Determination of monolayer permeability using Transwell assays. Doxycycline decreased IL-1β-induced microvascular endothelial cell monolayer hyperpermeability. IL-1β induced hyperpermeability significantly, compared to untreated control group in this model (*p < 0.05; n = 5). Pretreatment with Doxycycline mitigated this effect compared to IL-1β group (**p < 0.05; n = 5).

The determination of monolayer permeability of brain endothelial cells, using a suitable tracer, especially a fluorescent tracer, is a reliable indicator of barrier integrity and microvascular permeability in vitro. Interleukin 1-β treatment induced hyperpermeability in the HBMEC monolayers evidenced by the enhancement in the FITC-dextran flux across the monolayer/barrier (*p* < 0.05; n = 5; Fig. 3). Doxycycline treatment protected the monolayers from 1-β induced monolayer hyperpermeability (*p* < 0.05; n = 5; Fig. 3).

### Doxycycline attenuates TBI-induced microvascular hyperpermeability

**Figure 4:**
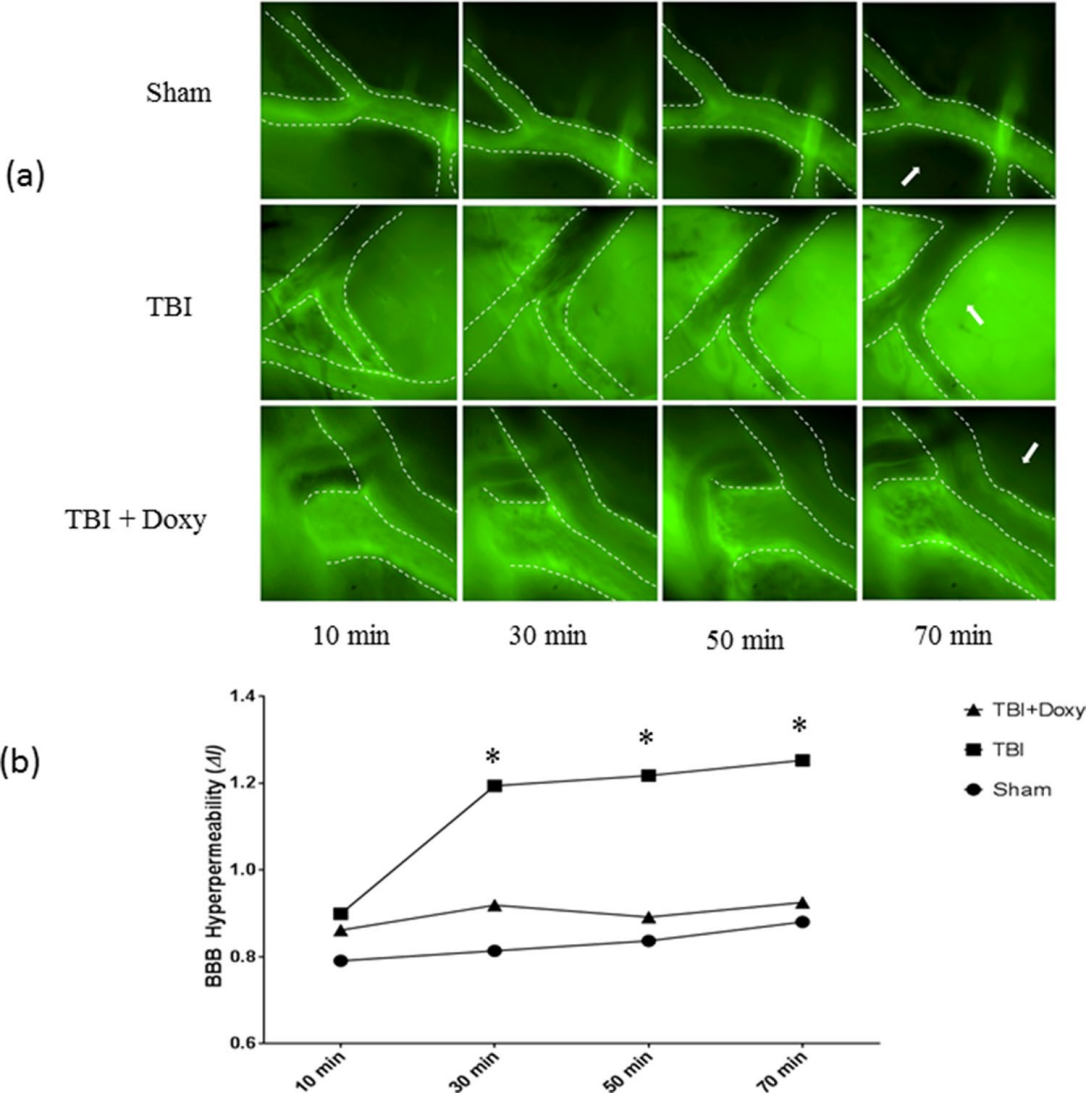
(**a**) Intravital microscopic imaging of mouse brain pial venules demonstrating changes in blood–brain barrier/microvascular permeability. Pial venules of 50-75 µm diameter were visualized at 40 × magnification. The imaging was started 10 min after injury or drug administration. Images and video were recorded every 20 min for comparison. Dotted lines have been added to better differentiate the pial venules. (**b**) Graphical plotting of BBB Permeability as Δ*I.* Two-way ANOVA showed no statistically significant difference among the groups at the initial time point of 10 min after injury. Increased microvascular hyperpermeability at 30, 50, 70 min of TBI was observed in comparison to sham (*p < 0.05; n = 5). TBI + Doxycycline group showed a significant decrease in hyperpermeability compared to TBI group (*p < 0.05; n = 5). No difference in Sham versus TBI + Doxycycline groups observed.

Intravital microscopy is a reliable imaging technique for evaluating the changes in vascular permeability in anesthetized/live lab animals. The data obtained from intravital microscopic imaging of pial venules of mouse is shown in Fig. 4. There was no significant difference among the groups at the initial time point of 10 min after injury (two-way ANOVA; *p* = 0.31). Microvascular permeability was calculated based on the formula, Δ*I* = 1 − (*I*i − *I*o)/*I*i where Δ*I* is the change observed in fluorescence intensity. Here, *I*i is the fluorescence intensity observed inside the vessel, and *I*o is the fluorescence intensity observed extravascularly. For the ease of visualization, the venules have been traced with a dotted white line (Fig. 4a, a graphical representation of the changes in BBB hyperpermeability (as a function of Δ*I*) is shown in Fig. 4b. TBI induced microvascular hyperpermeability at 30, 50, 70 min compared to sham group (*p* < 0.05; n = 5). The hyperpermeability observed following TBI, was decreased in TBI + Doxycycline group when compared to TBI group (*p* < 0.05; n = 5).

### Brain MMP-9 activity is increased in TBI and inhibited by doxycycline

**Figure 5:**
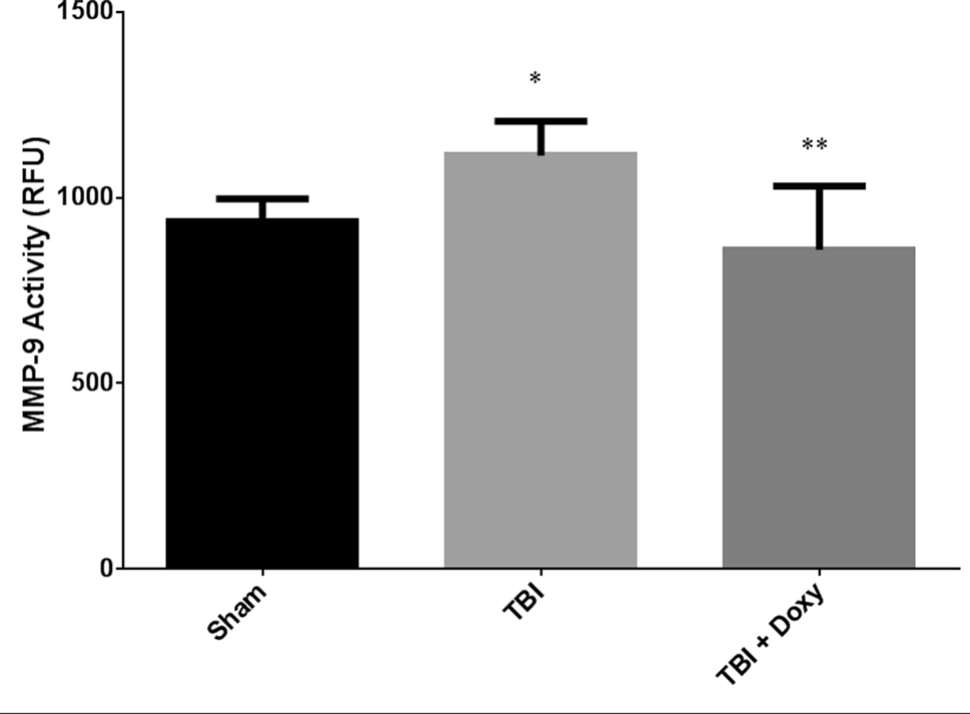
Fluorometric Measurement of MMP-9 Activity. Brain tissue from various groups was collected and processed for MMP-9 activity. Significant increase of MMP-9 activity was observed in TBI group compared to sham group (*p* < 0.05; n = 4). TBI + Doxycycline group showed a significant decrease in MMP-9 activity compared to TBI group (*p* < 0.05; n = 4). ‘*’ compared to sham. '**' compared to TBI.

The fluorometric assay of MMP-9 Activity conducted on brain tissue from mice in each experimental group showed statistically significant increase in MMP-9 enzyme activity in the TBI group when compared with Sham group (*p* = 0.008; n = 4). There was a statistically significant decrease in MMP-9 activity in TBI + Doxycycline group compared to TBI group without doxycycline treatment (*p* < 0.05; n = 4) (Fig. 5).

## Discussion

This study addresses an existing void in the treatment of secondary injury due to TBI and suggests efficacy of a pharmacologic therapy to attenuate BBB dysfunction following TBI. We used computational, cell, and small animal models in support of the use of doxycycline as an effective therapeutic to attenuate BBB hyperpermeability after traumatic brain injury.

Our pre-clinical study is novel in that it strongly implicates doxycycline as a viable therapeutic option to limit secondary BBB breakdown following TBI when established in clinical settings. Through in silico molecular docking studies, we have demonstrated that MMP-9 active site make strong interactions with doxycycline in a thermodynamically favorable manner. Although some previous studies from our lab and by others have suggested the cellular inhibition of MMP by doxycycline^16–18^, the current study shows a direct molecular interaction and inhibition, which has not been observed before. Our cellular models show that doxycycline protects tight junction integrity and prevents IL-1β-induced microvascular hyperpermeability in brain microvascular endothelial cells in vitro. Our small-animal model described here, further supports these findings as evidenced by reduced BBB dysfunction/microvascular hyperpermeability following TBI in mice who received doxycycline; also, doxycycline-treated animals had decreased MMP-9 activity in their brain tissue after TBI, giving credence to our computational studies.

Matrix metalloproteinase-9 was the main target of this study as it is well-established in the pathobiology of trauma and particularly traumatic brain injury^7,21^. Previous research has demonstrated enhanced levels of MMP-9 in the CSF and blood in human TBI subjects^21^. MMP-9 has also been implicated in BBB breakdown following ischemic stroke^22^. MMP-9 is a Zn^2+^ ion containing endopeptidase where the metal ion plays a significant role for its function and structural stability^15^. An effective MMP-9 inhibitor requires chelating the Zn^2+^ ion, binding with the backbone residues of the protease, and inhibition of the enzyme/protein activity. Previous studies have shown that MMP-9 small molecule inhibitors bind to the S1’ substrate binding pocket, close to the Zn^2+^ ion configuration^23^. Our docking studies revealed that the interaction of these MMP-9 key residues helps in positioning the doxycycline in S1’ pocket for its proper function. Doxycycline demonstrates a good docking score of −7.097 kcal/mol with some hydrogen and hydrophobic interactions and a coordinate bond with Zn^2+^ ion. A docking score less than zero implies a spontaneous, thermodynamically favorable reaction between the receptor and ligand. Specific MMP-9 inhibition in the S1’ pocket occurs by binding to LEU-222 and VAL-223 residues^23^. Our docking studies demonstrate a hydrophobic interaction of doxycycline towards VAL-223 residue and which in turn determines its specificity towards MMP-9. The present doxycycline interaction analysis confirms MMP-9 active site residues for its inhibition and Zn^2+^ ion chelation. Based on these results, we believe that the antagonism of MMP-9 is well-supported in our study. Postmortem brain tissue in the TBI affected hemisphere was shown to have increased MMP-9 enzyme activity after TBI. This MMP-9 activity was returned to sham levels with administration of doxycycline after TBI both on western blot analysis and enzyme activity assay in this tissue. These ex vivo findings reinforce the in silico data that doxycycline binds to the active site of MMP-9 to inhibit the deleterious effect and suggest that the protective effect of doxycycline occurred via preservation of BBB integrity.

Intravital microscopic imaging is a reliable tool to study real-time microvascular dynamics. The real-time BBB dysfunction and hyperpermeability was captured with intravital microscopy in our mouse model for TBI. It is important to note that the BBB dysfunction observed in this study were effectively mitigated with a one-time IV injection of doxycycline. This study evaluated hyperacute/acute changes in TBI after doxycycline treatment. Our results demonstrate that the greatest increase in BBB dysfunction and microvascular permeability occurred between ten and thirty minutes after traumatic brain injury. A major strength of our study was the selection of doxycycline that inhibits MMP-9, as a therapeutic agent. The tetracyclines including doxycycline exert their antibacterial effect mainly by binding to the bacterial ribosome and inhibiting protein synthesis^25^. Thus, the potential role of doxycycline other than inhibiting MMP-9, is also a subject of interest. Doxycycline possesses anti-inflammatory properties and possesses broad beneficial effects in the brain and improved survival in a model of *pneumococcal meningitis* in infant rats^20^. Particularly, doxycycline inhibits tumor necrosis factor alpha-converting enzyme that promotes inflammation, BBB disruption, and brain injury in conditions such as bacterial meningitis^20^. The major advantage of doxycycline is that it is an FDA-approved antibiotic with a well-established side effect profile (with known side effects such as headache, nausea, vomiting etc.) and without any major aide effects (20; 25). We sought to use a dose that was well within the therapeutic range of doxycycline. The dose of 20 mg/kg of doxycycline given to mice in this study when converted to human doses is 1.6 mg/kg which is well below the antimicrobial dose^24^. We utilized immunofluorescence localization of the tight junction-associated protein ZO-1 to observe the structural changes that takes place with the secondary injury in TBI in brain microvascular endothelial cells. Our results demonstrated that doxycycline-treated endothelial cells both had improved tight junction integrity in ZO-1 immunofluorescence localization and retained barrier function following exposure to IL-1β. In the immunofluorescence studies, we can visualize that the ZO-1 protein signal at the cell–cell junctions is significantly decreased and discontinuous. Previous studies from our lab demonstrated that ZO-1 expression (studied, by Western blot and RT-PCR) was not altered because of IL-1β treatment^14^. However, ZO-1 localization was diminished/discontinuous at the endothelial cell–cell junctions suggesting that the decrease in ZO-1 may not be due to changes in its protein expression but potential relocation from the inter-endothelial tight junctions to other cellular compartments^14^.

There are certain limitations that we recognize about this pre-clinical study. This study is a non-survival, small-animal experiment so the long-term outcomes have not been determined in these animals. The evaluation of doxycycline treatment following behavioral/memory challenges associated with TBI will be the subject of our future research. The endothelial monolayer permeability assay used in this study is a reliable in vitro test of barrier function, but it is limited as only the endothelial component of the BBB is represented and other cellular components of the neurovascular unit is not included. The short-term nature of the animal experiments with the increase in vascular permeability within thirty minutes of injury is not that easy to translate the optimal window to administer doxycycline in human head trauma subjects. Identifying the appropriate treatment dose and timing will be the focus of future research in this area.

In conclusion, we have demonstrated through post-injury TBI treatment that doxycycline may be a viable acute therapy for cerebral edema following TBI in a pre-clinical animal model; and the cellular/molecular mechanism of action is potentially through molecular inhibition of MMP-9 via directly binding to the active site of the enzyme and subsequent preservation of integrity of the BBB from MMP-9-based proteolytic breakdown of tight junction proteins. One of the clinical implications of this study is that, based on the timeframe of this study, early doxycycline could be administered by emergency medical services personnel at the scene or the medical team upon arrival of patients to the trauma bay^25^. It is our expectation that this study will lead to possible future clinical research and trials using doxycycline to restore BBB integrity and thereby alleviates the symptoms of cerebral edema in head injury patients.

## Materials and methods

### Molecular modeling/in silico docking studies

Molecular modeling and docking provide a thorough structural understanding of the strength of protein and ligand binding^26–28^. Using a suitable protein structure, the possible binding modes of a particular ligand as well as the strength of the interaction in terms of binding energy can be determined. Using Schrödinger software suite Glide module (Glide v6.5, Schrödinger, LLC, New York, NY) molecular docking of doxycycline to MMP-9 active site was performed. Glide uses a hierarchical docking strategy wherein the different ligand conformations of doxycycline are generated and then hierarchically tested for best fit with minimum energy at the MMP-9 active sites.

### Protein and ligand preparation

The catalytic domain of MMP-9 protein crystal structure was obtained from Protein Data Bank (PDB) with structure ID 4XCT having 1.3 Å resolution^29^. Protein preparation involves adding hydrogens, bond orders, and formal charges. Water molecules present in the active site were preserved. The geometry of protein structure was optimized, and energy minimized with the force field Optimized Potential for Liquid Simulations 2005 (OPLS-2005) using Refinement module to prevent the atomic level steric clashes in crystal structure. The structure of doxycycline was prepared using Ligand Preparation module of Schrödinger software. Hydrogen atoms were added to doxycycline and further structure was minimized using same force field at neutral pH.

### Grid generation and docking

The search space for docking was determined by picking the co-crystallized ligand interacting with the active site of MMP-9. Receptor grid generation module of Glide was used to generate the grid (search space). The x, y, z center coordinates of the grid were 18.082, −18.311, 19.012. MMP-9 requires Zn^2+^ ion as a cofactor for its enzymatic activity. There are two Zn^2+^ ions in MMP-9 enzyme, one Zn^2+^ ion aids in its catalytic activity while the other helps in maintaining the structure of the enzyme^30^. Binding of an inhibitor to the catalytic Zn^2+^ ion is responsible for enzyme activity inhibition. Tetracyclines are known to inhibit MMP-9 by Zn^2+^ ion chelation^31^ hence Zn^2+^ ion ligand binding constraint was considered in our computation during the grid generation step. Extra precession (XP) docking strategy was employed to dock doxycycline at the MMP-9 protein active site. Finally, the interactions between MMP-9 and doxycycline were visualized using PyMOL, a molecular graphics and modeling software^32^.

### Endothelial cell culture

Human brain microvascular endothelial cells (HBMECs; primary cultures) used in this study were obtained commercially (Cell-Systems, Kirkland, WA). The cells were first grown on fibronectin-coated culture dishes, using the HBMEC medium (ScienCell, Carlsbad, CA) in a laboratory cell culture incubator (95% O2, 5%CO2 at 37 °C). The cells were exposed to 0.25% trypsin–EDTA for dissociation. The dissociated cells were later grown on fibronectin-coated culture dishes, chamber slides or Transwell plates as required for various experiments. The rat brain microvascular endothelial cells (RBMECs) used in some of the experiments were obtained from Cell Applications, Inc. (San Diego, CA).

### Junctional protein localization by immunofluorescence

RBMECs were used for immunofluorescence localization of the tight junction proteins ZO-1. The cells were grown as a monolayer on chamber slides overnight. They were first exposed to a reduced serum medium (Opti-MEM); Life Technologies, Grand Island, NY). Following this, the cells were treated with doxycycline (20 µM) for 1 h) followed by treatment with interleukin-1β (IL-1β; 10 ng/mL) for two hours. Two sets of experiments were performed with four replicates each per group in an eight well/slide setting. Previous studies have shown that IL-1β increases MMP-9 expression significantly^33^. RBMECs were then fixed in paraformaldehyde (4%) followed by permeabilization with 0.5% Triton-X 100 (Sigma-Aldrich, Carlsbad, CA) for 15 min. This was followed by blocking with bovine serum albumin (2%) (Sigma-Aldrich) and overnight incubation with a primary antibody (anti-rabbit ZO-1 (catalogue #617300; 1:150; ThermoScientific, Waltham, MA). This was followed by incubation with a secondary antibody tagged with FITC (1:150; Santa Cruz, Dallas, TX) for 60 min. VECTASHEILD® containing DAPI was used as an antifade mounting media (Vector Laboratories, Burlingame, CA). ZO-1 immunofluorescence was visualized using a confocal laser scanning microscope (Olympus Fluo-view) and the images were quantified using ImageJ software (National Institutes of Health) at 10 distinct locations in each microscopic field recorded and the means compared.

### Endothelial cell monolayer permeability

HBMECs were grown to confluent monolayers on fibronectin coated Transwell inserts (Corning Life Sciences, Lowell, MA). Sixty minutes before each experiment, cell culture media was changed/ replaced with phenol red-free cell culture media (DMEM; Life Technologies) to avoid the interference of phenol red with during fluorescence measurement. Wells of the Transwell plates were divided into four different groups (n = 5). Group one consisted of an untreated control group and the group two was consisted of a doxycycline treated group. The third group received IL-1β treatment (for inducing monolayer hyperpermeability) and the fourth group consisted of a doxycycline + IL-1β group. Two sets of experiments were performed with five replicates per group. Each experimental group were included in a 12-well/plate monolayer plate setting. In the doxycycline treated cells, wells were pretreated with 20 µM doxycycline). This concentration was previously demonstrated to possess anti-MMP-9 activity in cellular studies in vitro^34,35^. Endothelial monolayer permeability was induced in the IL-1β groups (10 ng/mL) with incubation for two hours. FITC-dextran (5 mg/mL; Sigma-Aldrich) was applied to the luminal (upper) chambers of the Transwell and was allowed to equilibrate through the monolayer between the upper and lower chambers for thirty minutes. Cell culture media samples were obtained from the abluminal (lower) chamber of the Transwell and measured by a fluorometric plate reader (excitation 494 nm/emission 520 nm) to quantitate FITC-dextran permeability across the monolayer barrier as a marker of permeability.

### Animals

All experiments conducted in this study utilized male C57BL/6 mice (18–25 g) purchased from The Jackson Laboratories (Bar Harbor, MA). The animals were maintained at the institutional (Texas A&M Health Science Center College of Medicine/Baylor Scott & White Research Institute) animal facility located on the campus. A 12:12 h dark/light cycle was maintained and were given with food and water ad libitum. The room temperature of the facility was maintained at 25° ± 2 °C. Institutional Animal Care and Use Committee (IACUC) of the Texas A&M Health Science Center College of Medicine and Baylor Scott & White Research Institute approved all surgical/experimental procedures. The animal facility was approved by the Association for Assessment and Accreditation of Laboratory Animal Care International and was in accordance with the National Institutes of Health guidelines. The animals were anesthetized with 30% urethane (Sigma-Aldrich; 2 mL/kg body weight) given intraperitoneally. They were constantly monitored by a researcher till the completion of the experimental period (up to two hours following traumatic brain injury). This study was terminal in nature, and we didn’t observe any unexpected deaths of mice during the study period. The study is reported in accordance with ARRIVE guidelines.

### Controlled cortical impact model of TBI

A controlled cortical impact model of TBI was used in this study. For inducing traumatic brain injury in mice, a midline incision was made exposing the sagittal suture, bregma and lambda. A craniotomy (5–6 mm in diameter) was performed over the right hemisphere, between lambda and bregma with a pedal operated micro drill. Sham mice received craniotomy alone. The TBI group were inflicted with brain injury using a controlled cortical impactor after craniotomy. This method utilizes the Benchmark™ Stereotaxic Impactor (Leica Biosystems Inc., Buffalo Grove, IL). After craniotomy procedure, the animals were placed on the stereotaxic frame. An impactor probe of 4 mm diameter was selected to impact the exposed portion of the brain. The impactor settings for moderate TBI used in this study are: 2 mm depth, 0.55 m/second velocity and 100 ms contact time^36^.

### Intravital microscopy

Intravital microscopic imaging is a reliable microscopic tool that permits visualization and imaging of microcirculation in real-time. Mice undergoing intravital microscopic imaging were divided into the following groups (n = 5/group): Sham group, TBI group, and TBI + Doxycycline group. Mice were anesthetized and the surgical procedures were followed as discussed earlier. Ten minutes after inflicting TBI, the TBI + Doxycycline group received a single dose of doxycycline hyclate (20 mg/kg; Sigma-Aldrich; via tail vein). A cover glass (No. 0 glass 5 mm; Warner Instruments, Hamden, CT) was then positioned over the craniotomy window and the animal was placed on a custom designed platform located on the stage of the microscope (Nikon E600, Tokyo, Japan). Temperature was maintained at 37 °C with a thermal pad throughout the duration of the experiment. The mice received a bolus of FITC-dextran-10 kDa (0.1 mL of 50 mg/mL: Sigma-Aldrich) via tail vein, immediately before TBI. Pial vessels of 50 to 75 µm identified for analysis using a Nikon 40× objective (Nikon Instruments, Inc., Natick, MA). Images were captured at 10-, 30-, 50-, and 70-min following injury. Images were obtained with a Photometrics Evolve Camera (Roper Scientific, Tucson, AZ). The images were captured digitally and analyzed using Nikon NIS Element Software.

### MMP-9 activity assessment ex vivo

MMP-9 activity was measured using a SensoLyte® 490 MMP-9 Fluorometric Assay Kit (Anaspec, Fremont, CA). The assay was capable of detecting MMP-9 activity in a variety of biological samples, using an EDANS/DabcylPlus™ fluorescence resonance energy transfer (FRET)5 peptide. For the assay, the brain tissue obtained from the various groups of mice (Sham, TBI, TBI + Doxycycline; n = 4) were collected and the injured hemispheres of the cerebrum were selected for the analysis. The brain tissue was homogenized using a homogenizer in the specified assay buffer provided in the kit. Homogenized tissue samples were collected and following protein estimation, 100 µg protein of protein from each sample, were taken in each well. This was followed by treatment with 4-aminophenylmercuric acetate (APMA) and incubated in the dark for 2 h to activate the pro-MMPs. Following this, MMP-9 substrate was added and incubated for another 30 min in dark. Fluorescence was measured in a plate reader at excitation 490 nm and emission 520 nm. The enzyme activity was calculated and expressed as relative fluorescence units (RFU) and plotted on the Y-axis.

### Statistical analysis

All experimental data obtained from various experiments described in this study are presented as mean ± SEM. Statistical analysis was conducted with GraphPad Prism 7 with analysis of variance (ANOVA) or paired *t* test whenever required. A ‘*p*’ value less than 0.05 was taken as statistically significant. For ANOVA a finding of statistical significance followed by Bonferroni’s or Tukey’s post hoc multiple comparison analysis where performed.
